# Robotic-assisted versus open resection of pulmonary sequestration: a retrospective cohort study. RATS surgery for pulmonary sequestration

**DOI:** 10.1007/s11748-025-02172-9

**Published:** 2025-06-24

**Authors:** Henrike Deissner, Alessio Campisi, Raffaella Griffo, Benedikt Niedermaier, Thomas Muley, Michael Allgäuer, Hauke Winter, Martin E. Eichhorn

**Affiliations:** 1 Department of Thoracic Surgery, Thoraxklinik, Heidelberg University Hospital, Roentgenstraße 1, 69126 Heidelberg, Germany; 2 Thoracic Surgery Unit, Cardiovascular and Thoracic Department, University and Hospital Trust-Ospedale Borgo Trento, Verona, Italy; 3 Translational Lung Research Center Heidelberg, German Center for Lung Research (DZL), Heidelberg, Germany; 4 Section Translational Research (STF), Thoraxklinik, Heidelberg University Hospital, Heidelberg, Germany; 5 Institute of Pathology, Heidelberg University Hospital, Heidelberg, Germany

**Keywords:** Robotic, Minimally invasive, Pulmonary sequestration, Thoracotomy

## Abstract

**Background:**

Pulmonary sequestration (PS) is a rare congenital lung malformation often requiring surgical resection due to recurrent infections or hemoptysis. Traditionally treated via open thoracotomy, recent advancements have made minimal-invasive approaches like robotic-assisted thoracoscopic surgery (RATS) increasingly viable. This study compares outcomes between RATS and open resection for PS in a high-volume center.

**Methods:**

In this retrospective cohort study, 23 adult patients who underwent surgical resection of PS between 2010 and 2023 were analyzed. Fifteen patients were treated via open thoracotomy (THKT), while eight underwent RATS using the DaVinci-X system. We compared preoperative findings, intraoperative variables, and postoperative outcomes.

**Results:**

The patients in the RATS group were younger (median age: 36 vs 47 years) and had a shorter median hospital stay (5 vs 10 days, *p* < 0.001) compared to the THKT group. The RATS group also experienced earlier chest drainage removal (3 vs. 4 days, *p* = 0.016). However, the median duration of surgery was longer for RATS (118 vs. 75 min, *p* = 0.018). A trend towards less postoperative complications was observed in the RATS group (33% vs. 0%).

**Conclusions:**

RATS provides a safe and effective alternative to open surgery for PS resection, with benefits including reduced hospital stay and earlier chest tube removal. Despite longer operative times, the minimally invasive approach may offer enhanced recovery and fewer complications. Continued accumulation of experience with RATS is likely to improve operative efficiency, making it a valuable option in the surgical management of pulmonary malformations.

**Supplementary Information:**

The online version contains supplementary material available at 10.1007/s11748-025-02172-9.

## Introduction

Pulmonary sequestration (PS) is an uncommon congenital lung malformation, characterized by non-functional pulmonary tissue with aberrant arterial supply, often arising directly from the aorta. The condition typically presents in childhood but may remain clinically silent until adulthood, where it often manifests through recurrent pulmonary infections or hemoptysis. Surgical resection is the treatment of choice due to the potential for significant, yet non-specific, symptoms and the risk of complications such as infection or hemorrhage [1, 2].

Traditionally, PS resection has been performed through open thoracotomy, a method that, while effective, is associated with considerable morbidity, including prolonged recovery times and increased postoperative pain [3]. The advances in minimally invasive surgery (MITS), specifically video-assisted thoracoscopic surgery (VATS) and more recently, robotic-assisted thoracoscopic surgery (RATS), offer alternatives that may reduce these risks [4, 5]. RATS, with its high-definition 3D visualization and enhanced dexterity provided by robotic instruments, presents a promising approach for the complex anatomy and potential adhesions associated with PS. Nevertheless, comparative data on the outcomes of RATS versus traditional open surgery for PS remain limited, with most existing literature focusing on case reports and small series [6–15].

In this study, we retrospectively compared the perioperative outcomes of patients who underwent RATS or open thoracotomy for PS resection at a high-volume thoracic surgery center. Our aim is to assess whether the advantages of RATS translate into improved clinical outcomes, thereby establishing it as a viable alternative to open surgery for pulmonary malformations.

## Patients and methods

This retrospective cohort study was conducted at the Thoraxklinik Heidelberg University. We included all adult patients who underwent surgical resection for PS between January 2010 and August 2023. The patients were divided into two groups: those who underwent open thoracotomy (THKT) from 2010 to 2019, and those who underwent RATS using the DaVinci-X system by Intuitive Surgical, Sunnyvale, CA, USA from 2019 to 2023, following the introduction of robotic surgery at our institution. Three patients who underwent VATS during the study period (one of whom required conversion to open surgery due to severe adhesions) were excluded because of the small size of the cohort. The institutional review board of the hospital approved the study (No. S-089/2018, 01.03.2018). The study was performed in line with the principles of the Declaration of Helsinki. The paper was written according to the STROCSS criteria (Strengthening the reporting of cohort studies in surgery). Its checklist is provided as Supplementary File.1 [16].

**Fig. 1:**
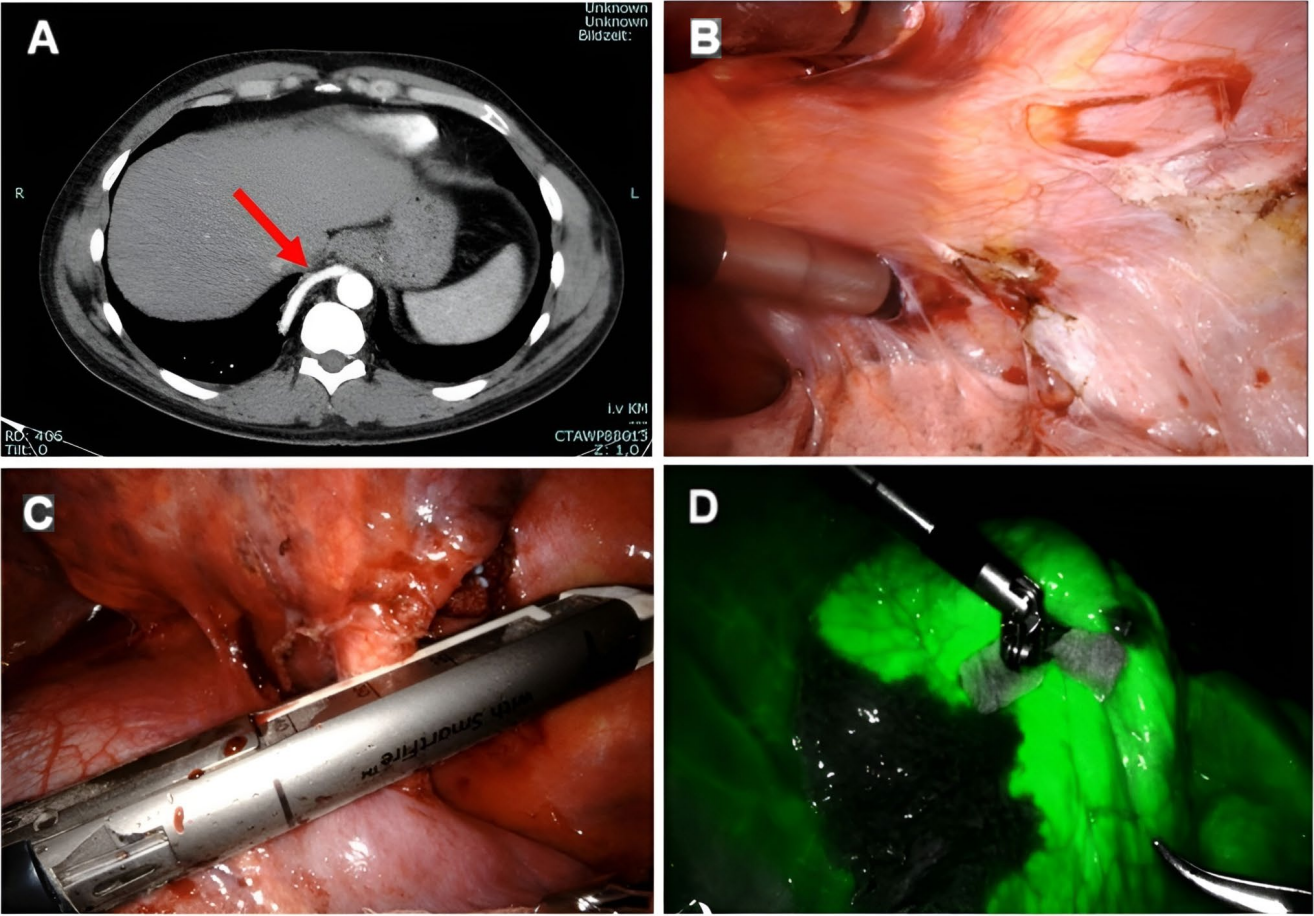
**A** CT scan showing a right-sided intralobar sequestration with an arterial vessel originating from the descending aorta; **B** Extensive adhesiolysis; **C** Vessel ligation using a robotic stapler; **D** Usage of ICG for parenchymal margin identification

**Fig. 2:**
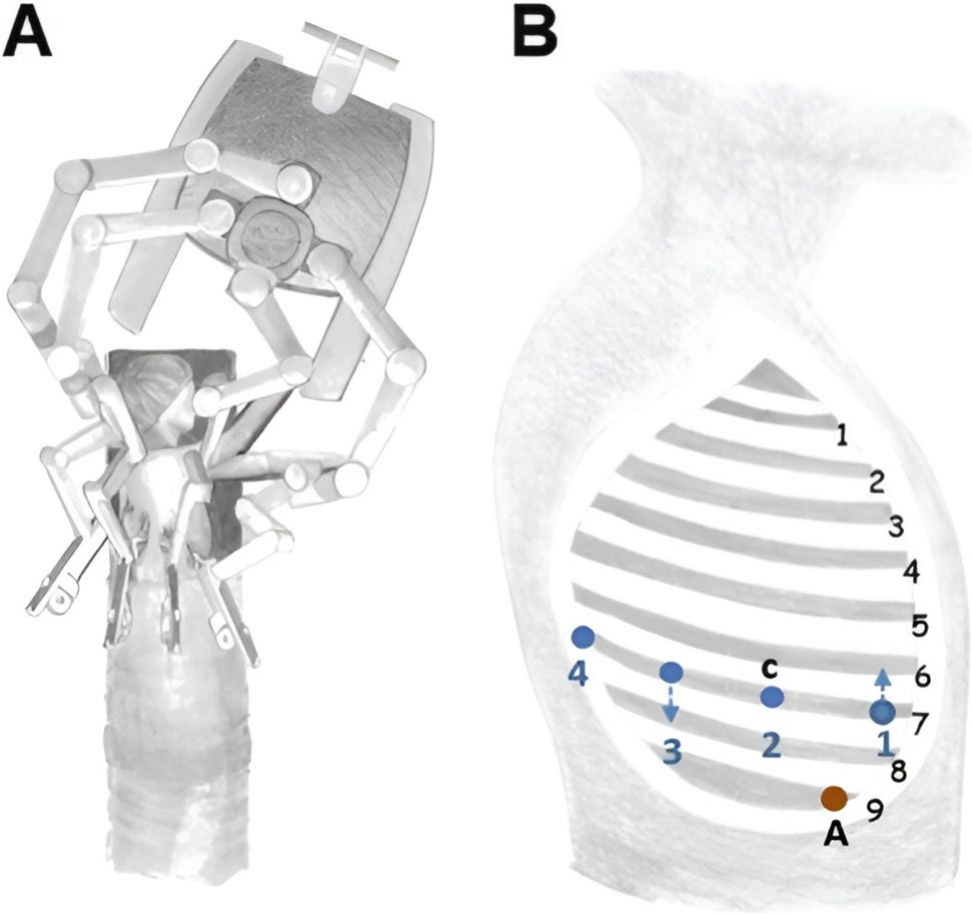
Port placement and intraoperative robotic setup

**Fig. 3:**
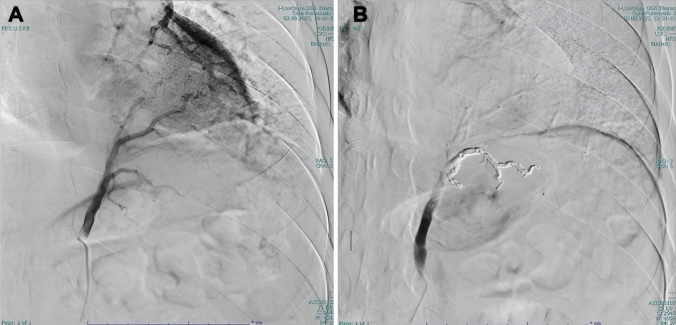
**A** Angiographic identification of the arterial branch of the PS with an arterial vessel originating from the celiac tripod; **B** Coiling of the vessel

All patients were managed according to institutional protocols, which included preoperative imaging (Fig. 1A), bronchoscopy, and antibiotic prophylaxis. For RATS, the patients were positioned in lateral decubitus with slight flexion of the operating table to widen the intercostal spaces. The DaVinci-X system patient cart was docked from the cranial side of the patient. Port placement was standardized as follows: the camera port was positioned at the VII intercostal space (ICS) at the mid-axillary line; the second robotic port (12 mm) at the VII ICS anterior axillary line; the third robotic port (12 mm) at the VII-VIII ICS posterior axillary line; and the fourth robotic port (8 mm) at the VII ICS posterior scapular line. Additionally, a 15 mm utility/assistant port was placed at the IX ICS between the camera and anterior ports (Fig. 2). CO₂ insufflation was initiated after the placement of the camera port to enhance exposure and facilitate lung collapse. A four-arm robotic technique was employed, using the following instruments: a curved bipolar dissector, a bipolar fenestrated forceps and a Tip-Up Fenestrated Grasper. An assistant surgeon operated through the utility port for stapling, suction, and specimen retrieval. Intraoperatively, extensive adhesiolysis was frequently necessary due to recurrent infections (Fig. 1B). Typically, the aberrant artery arising from the systemic circulation was closed using a standard stapler (Covidien, Endo GIA™ universal Roticulator™ white reload 30 mm) inserted through the assistant port or the SureForm 45 Curved-Tip stapler (Intuitive Surgical, Sunnyvale, CA) (Fig. 1C) and indocyanine green (ICG) fluorescence was employed to delineate the sequestration area (Fig. 1D), guiding the extent of resection. Hem-o-Lok clips were used to close vessels smaller than 4 mm. Alternatively, when the risk of bleeding was considered high, the feeding vessel was preoperatively coiled the day before surgery by an interventional radiologist (Fig. 3).

In the THKT group, open thoracotomy was performed with similar preoperative and intraoperative management, excluding the use of ICG fluorescence. The extent of resection was determined based on macroscopic evaluation of the lung tissue and vascular supply.

### Statistical analysis

We collected data on patient demographics, preoperative symptoms, intraoperative variables, and postoperative outcomes. Continuous variables were reported as medians with interquartile ranges (IQR), while categorical variables were reported as numbers and percentages. Statistical comparisons between groups were made using the Mann–Whitney U test for continuous variables and the Chi-square test for categorical variables. A p-value of less than 0.05 was considered statistically significant. The statistical analysis was performed using Prism (version 8.2.1, GraphPad Software Inc., Boston, USA).

## Results

A total of 23 patients (Table 1) were included in the study: 15 in the THKT group and 8 in the RATS group (Table 2). The median age was 36 years (IQR 33–45) in the RATS group and 47 years (IQR 44–54) in the THKT group (*p* = 0.672). The preoperative symptoms were similar between groups, with the majority presenting with recurrent infections (75.0% in the RATS group vs. 53.3% in the THKT group). Hemoptysis was observed in 25% of the RATS group and 13.3% of the THKT group.

**Table 1:** Comparison of RATS and open approach groups

	RATS (*n* = 8)	Open resection (*n* = 15)	p-Wert
Sex			
Male	3 (37.5%)	7 (46.7%)	0.672^b^
Female	5 (62.5%)	8 (53.3%)	
Age	36 (30–45)	46 (43–54)	0.100^a^
Location			
RLL	2	2*	0.482^b^
LLL	6	13	
Anatomy			
Extralobar	3 (37.5%)	2 (13.3%)	0.180^b^
Intralobar	5 (62.5%)	13 (86.7%)	
Adhesions	7 (87.5%)	12 (80.0%)	0.651^b^
Symptoms			
Infection	6 (75.0%)	2 (13.3%)	0.003^b^*
Hemoptysis	2 (25.0%)	8 (86.7%)	0.191^b^
Dyspnea	3 (37.5%)	2 (13.3%)	0.180^b^
Pain	2 (25.0%)	4 (26.7%)	0.930^b^
Complications	0 (0.0%)	5 (33.3%)	0.064^b^
Pneumonia	0 (0.0%)	1 (6.7%)	
Pleural Effusion	0 (0,0%)	1 (6.7%)	
Bleeding	0 (0.0%)	1 (6. 7%)	0.637^b^
Lung gangrene	0 (0.0%)	1 (6.7%)	
Pseudoarthrosis	0 (0.0%)	1 (6.7%)	
Transfusion	0 (0.0%)	1 (6.7%)	0.455^b^
Surgical revision	0 (0.0%)	3 (20.0%)	0.175^b^
Skin-to-skin-time (min)	118 (89–178)	75 (55–90)	0.015^a^*
Type of resection			
Wedge	3 (37.5%)	5 (33.3%)	
Segmentectomy	4 (50.0%)	9 (60.0%)	0.851^b^
Lobectomy	1 (12.5%)	1 (6.7%)	
ICU Days (days)	1 (1–1)	1 (1–1)	0.126^a^
Chest drainage Duration (days)	3 (2–3)	4 (3–5)	0.014^a^*
LOS (days)	5 (4–5.75)	10 (8–11)	< 0.001^a^*

Data are presented as median (interquartile range) or n (%)

*RLL* Right lower lobe, *LLL* Left lower lobe, *ICU* Intensive care unit, *LOS* Length of hospital stay

^*^p < 0.05

^a^Mann–Whitney *U* test

^b^Chi-square test

**Table 2:** Characteristics of patients treated by RATS

	Patient 1	Patient 2	Patient 3	Patient 4	Patient 5	Patient 6	Patient 7	Patient 8
Age (years)	36	29	34	55	45	28	35	44
Sex (M/F)	f	f	m	f	f	m	m	f
Symptoms								
Infection	x	x	–	x	x	x	x	–
Hemoptysis	x	–	–	–	x	–	–	–
Dyspnea	–	–	x	x	–	–	–	x
Pain	–	x	–	–	–	x	–	–
Others	–	–	–	–	–	–	–	–
Location	RLL	LLL	RLL	LLL	LLL	LLL	LLL	LLL
extralobar	–	x	–	x	–	–	–	x
intralobar	x	–	x	–	x	x	x	–
Artery	thoracic aorta	thoracic aorta	thoracic aorta	thoracic aorta	thoracic aorta	thoracic aorta	coeliac trunk	thoracic aorta
Venous Drainage	pulmon. veins	azygos vein	pulmon. veins	azygos vein	pulmon. veins	pulmon. veins	pulmon. veins	azygos vein
Adhesions	x	x	–	x	x	x	x	x
ICG	x	x	x	x	x	x	–	x
Preinterventional coiling	–	–	–	–	–	–	x	–
Complications	–	–	–	–	–	–	–	–
Thoracotomy	–	–	–	–	–	–	–	–
Console time (min)	47	79	42	73	181	84	140	114
Skin-to-skin-time (min)	86	112	74	98	208	124	190	142
Extend of resection								
Wedge	x	–	x	–	–	–	–	x
Segmentectomy	–	x	–	x	x	x	–	–
Lobectomy	–	–	–	–	–	–	x	–
ICU Days (days)	–	1	1	1	1	1	1	1
Chest tube duration (days)	2	2	2	3	3	3	6	3
LOS (days)	3	5	4	6	5	4	7	5

*m* male, *f* female, *RLL* Right lower lobe, *LLL* Left lower lobe, *ICG* Indocyanine green, *ICU* Intensive care unit, *LOS* Length of hospital stay

In the RATS group, the median console time was 82 min (IQR 67–121), with a median skin-to-skin time of 118 min (IQR 95–154). The adhesions were present in 87.5% of cases and the median blood loss was 40 ml (IQR 28–53). In contrast, the THKT group had a shorter median operative time of 75 min (IQR 58–85) (*p* = 0.015), with adhesions requiring release in 80% of the cases.

No significant differences were observed in the location of the PS (right or left lower lobe, p = 0.482), type of PS (extralobar or intralobar, *p* = 0.180), or type of resection performed (wedge resection, segmentectomy, or lobectomy, *p* = 0.851).

The RATS group experienced a significantly shorter median hospital stay (5 days, IQR 4–5) compared to the THKT group (10 days, IQR 8–11, *p* < 0.001). The chest drainages were removed earlier in the RATS group (median: 3 days, IQR 2–3) than in the THKT group (median 4 days, IQR 3–5, *p* = 0.016). No complications or conversions to open surgery occurred in the RATS group, whereas in the THKT group a 33% complication rate was observed, with 20% requiring revision surgery or interventional therapy.

## Comment

Our study contributes to the growing body of evidence that supports the use of RATS as a feasible and effective method for the resection of PS. When compared with traditional open thoracotomy, RATS offers several distinct advantages, particularly in terms of postoperative recovery and surgical precision. The significantly shorter hospital stay and earlier chest tube removal observed in our RATS cohort underscore a more rapid postoperative recovery, which is not only beneficial for patient outcomes but also has important implications for reducing healthcare costs. The absence of postoperative complications in the RATS group, despite the inherently longer operative times associated with the learning curve and technical intricacies of robotic surgery, further underscores the safety and viability of this approach.

One of the most noteworthy advantages of RATS highlighted in our study is the use of ICG fluorescence. This technique facilitated precise delineation of the sequestration area, allowing for maximal preservation of healthy lung parenchyma [17]. Such precision is particularly valuable in PS cases, where the risk of unnecessary resection of functional lung tissue can be mitigated. The ability to preserve lung parenchyma is crucial in maintaining postoperative pulmonary function, especially in cases involving complex anatomy or significant adhesions. The enhanced dexterity and superior visualization offered by the robotic system play a pivotal role in performing meticulous adhesiolysis, which is often required due to recurrent infections. This is a critical advantage over traditional thoracotomy, where extensive adhesiolysis can be more challenging and less precise.

Our findings align with existing literature that has documented the benefits of MITS, such as VATS, over open thoracotomy [4, 5]. However, our study extends these observations by demonstrating the additional benefits offered by RATS, particularly in the context of complex cases. While the longer operative time observed in the RATS group is a notable consideration, it can be attributed not only to the current stage of the learning curve associated with robotic surgery but also to the inherently more meticulous and precise dissection enabled by the robotic platform [18]. Moreover, the additional time required for docking the robotic system must be considered, which may partially compensate for the thoracotomy incision and closure time in open surgery. Importantly, pulmonary sequestration is a rare condition, and even in high-volume centers, opportunities to gain surgical experience with this disease remain limited. This further emphasizes the value of a precise and safe surgical approach such as RATS, which likely contributed to the lower complication rate observed in our robotic cohort. As robotic techniques continue to evolve and surgical experience increases, we anticipate that operative times will decrease, further enhancing the efficiency and appeal of RATS for PS resection. Furthermore, the earlier removal of chest drains observed in the RATS group may reflect trends reported in the MITS literature, including both VATS and RATS. Several studies have demonstrated that MITS is generally associated with shorter hospital stays compared to open thoracotomy [4, 19]. Although most reports do not explicitly provide data on chest tube duration, reduced length of stay may indirectly suggest earlier chest drain removal. In RATS, enhanced visualization and refined tissue handling may help minimize postoperative air leaks and fluid production, potentially facilitating earlier drain management. Nevertheless, the decision to remove chest drains remains multifactorial and is often influenced by surgeon preference, institutional protocols, and factors such as the volume and character of fluid output. Thus, while our findings suggest a trend toward earlier drain removal in the RATS group, this observation should be interpreted with caution due to the small sample size and lack of direct comparative data.

While our study provides valuable insights, several limitations should be acknowledged. First, the retrospective nature of the study may introduce selection bias, as patients who underwent RATS may have been preselected based on factors that made them more suitable candidates for this minimally invasive approach. Additionally, the relatively small sample size limits the generalizability of our findings, and the results may not be applicable to all patient populations, particularly those with more complex or atypical presentations of PS. Another limitation is the inherent learning curve associated with robotic surgery, which may have contributed to the longer operative times observed in our study. The variation in surgical expertise among the operating surgeons could also have influenced the outcomes, making it difficult to fully assess the efficiency and reproducibility of RATS across different clinical settings. Finally, the lack of long-term follow-up data in our study prevents us from drawing definitive conclusions about the durability of the outcomes and the potential for late complications or recurrence.

## Conclusion

Our study suggests that RATS is a safe and effective alternative to open thoracotomy for the resection of PS, with clear advantages including shorter hospital stays, earlier chest tube removal, and a potentially lower complication rate. While the current requirement for longer operative times remains a challenge, the overall benefits of RATS, particularly in terms of patient recovery and the preservation of healthy lung tissue, make it a compelling option in the management of this rare congenital lung condition. Future studies with larger cohorts and longer follow-up periods are warranted to confirm these findings and to further refine the application of robotic surgery in thoracic procedures.

## Supplementary Information

Below is the link to the electronic supplementary material.

Supplementary file1 (DOCX 32 KB)

## Data Availability

The data underlying this article will be shared on reasonable request to the corresponding author.
